# Roadmap for the use of base editors to decipher drug mechanism of action

**DOI:** 10.1371/journal.pone.0257537

**Published:** 2021-09-21

**Authors:** Estel Aparicio-Prat, Dong Yan, Marco Mariotti, Michael Bassik, Gaelen Hess, Jean-Philippe Fortin, Andrea Weston, Hualin S. Xi, Robert Stanton

**Affiliations:** 1 Simulation and Modelling Sciences, Pfizer, Cambridge, Massachusetts, United States of America; 2 Internal Medicine Research Unit, Pfizer, Cambridge, Massachusetts, United States of America; 3 Division of Genetics, Department of Medicine, Brigham and Women’s Hospital, Harvard Medical School, Boston, Massachusetts, United States of America; 4 Facultat de Biologia, Departament de Genètica, Microbiologia i Estadística, Universitat de Barcelona, Barcelona, Catalonia, Spain; 5 Department of Genetics, Stanford University, Palo Alto, California, United States of America; 6 Discovery Sciences, Pfizer, Groton, Connecticut, United States of America; Hirosaki University Graduate School of Medicine, JAPAN

## Abstract

CRISPR base editors are powerful tools for large-scale mutagenesis studies. This kind of approach can elucidate the mechanism of action of compounds, a key process in drug discovery. Here, we explore the utility of base editors in an early drug discovery context focusing on G-protein coupled receptors. A pooled mutagenesis screening framework was set up based on a modified version of the CRISPR-X base editor system. We determine optimized experimental conditions for mutagenesis where sgRNAs are delivered by cell transfection or viral infection over extended time periods (>14 days), resulting in high mutagenesis produced in a short region located at -4/+8 nucleotides with respect to the sgRNA match. The β2 Adrenergic Receptor (B2AR) was targeted in this way employing a 6xCRE-mCherry reporter system to monitor its response to isoproterenol. The results of our screening indicate that residue 184 of B2AR is crucial for its activation. Based on our experience, we outline the crucial points to consider when designing and performing CRISPR-based pooled mutagenesis screening, including the typical technical hurdles encountered when studying compound pharmacology.

## Introduction

The use of CRISPR (Clustered Regularly Interspaced Short Palindromic Repeats) with Cas9 (CRISPR associated protein 9) has proven to be a revolutionary technology enabling the rapid introduction of genetic perturbations at targeted genomic regions. Initially, CRISPR was primarily used as a tool for specific and efficient gene knockout [1–3]. Since then, CRISPR-based methods have been developed for multiple research purposes, including gene activation/inhibition studies [4], genetic screens [5], cell line engineering [6], and imaging [7] among others. Moreover, various applications of CRISPR emerged both for diagnostics [8] and for therapy (reviewed in [9]), which recently yielded the first positive results in clinical trials [10].

Base editors were initially developed in 2016 fusing a base editor enzyme (such as a deaminase) to the inactive form of Cas9 (dCas9) or alternatively to a nickase (creating single strand breaks) [11–14]. Cytosine base editors and adenine base editors allow the introduction of point mutations without producing a double strand break on the DNA, creating a potential for mutagenesis studies on endogenous proteins. The mutagenesis location is determined by the sgRNA used. The efficiency of base editors has improved greatly over time, but remains dependent on the base editor and the cell types used (reviewed in [15]). Recent advancements in the base editing field have included narrowing of the base-pair window within the gene that undergoes mutagenesis [16], reduction of off-targets [17], and increasing the protospacer adjacent motif (PAM) compatibility for greater coverage of DNA that is amenable to base editing [18]. Additionally, “prime editing” has been introduced, which directly rewrites information on a target DNA site: here, the effector system consists of an impaired Cas9 fused with a reverse transcriptase, and a peculiar guide RNA specifies both the target site and the desired edit [19].

Mechanism of action (MoA) studies play key roles in drug discovery. They are important for identifying potential safety liabilities and can assist in the approval of new drugs initially identified through phenotypic screening. Knowing the MoA of a small molecule can also help with drug optimization, as specific receptor interactions can be targeted. Traditional MoA studies are performed by exogenous expression of multiple plasmids, including the protein of interest by mutating residues one at a time (e.g., by alanine scanning [20]), but are not conducted on the endogenous protein. Recent advances in base editing may enable novel high throughput, targeted and multiplexed mutagenesis workflows to efficiently probe the role of specific residues within virtually any protein families.

Here, we show how a CRISPR-based screening approach can improve MoA studies, reducing time and costs and also allowing direct targeting of endogenous proteins. We set up a high-throughput targeted multiplexed mutagenesis workflow, in which a reporter system is employed with fluorescence-activated cell sorting and Next Generation Sequencing (NGS) to monitor the functional implications of mutations.

We explore the utility of base editors in a typical drug discovery setting using a pooled CRISPR approach to identify key residues driving small molecule pharmacology of a G-protein coupled receptor (GPCR). GPCRs represent the largest transmembrane receptor family encoded by the human genome and a successful drug target class [21]. Various types of GPCRs modulators including agonists, antagonists and allosteric modulators are being developed as therapeutics. Despite successes in developing several GPCR-based drugs, the identification of safe and efficacious modulators remains a challenging process. The development of novel strategies to dissect the structure-function relationships underlying the complex mechanisms by which ligands bind and activate GPCRs could markedly accelerate the development of effective therapeutics. The fact that emerging base editor technologies allow manipulation of endogenously-expressed proteins offers a unique opportunity to study GPCRs in a more native context with more appropriate expression levels and physiological context [22].

Here we use base editors in a pooled format, coupled with a functional selection method and next generation sequencing (NGS) to help elucidate the mechanisms of GPCR pharmacology and ligand-protein interactions in an endogenous cellular context. Additionally, we built on our experience to outline the driving principles, challenges and limitations of this approach, laying out a roadmap for future MoA studies.

## Materials and methods

### Cell lines

HEK293T cells with stable GFP and mCherry were obtained from the Bassik lab at Stanford [13]. HEK293T wild type cells were obtained from ATCC (CRL-3216). All cells were cultured with DMEM, 10% FBS, penicillin-streptomycin and L-glutamine. For drug selection, we used 2ng/μL puromycin, 4ng/μL blasticidin, and 300 μg/mL zeocin.

Transfection was performed with Lipofectamine 2000 (ThermoFisher) or Lipofectamine CRISPRMAX Cas9 Transfection reagent (ThermoFisher). Electroporation was performed using Neon transfection system (ThermoFisher).

### Plasmids for modified CRISPR-X

pGH389_AID*-dCas9-BlastR was obtained from the Bassik lab at Stanford [13]. We employed the direct fusion of AID* to the N-terminus of dCas9 because it displayed improved editing efficiency compared to the MS2 recruitment system (Hess et al., in preparation). This enhanced efficiency occurred within a smaller 20 bp window. On average, we expect that bases no longer targeted by the reduced window could be targetable by nearby sgRNAs, given that the expected frequency of an NGG PAM is 8 bp. Therefore, we chose to use this configuration of the CRISPR-X base editor. sgRNAs were cloned in plasmid #234 sgRNA-PuroR (no MS2) (pGH020 with Ef1-puro) since the MS2 hairpins were not required for this editor. sgRNAs against GFP were sgGFP10 and sgGFP1 from [13]. Oligonucleotides with overhangs compatible with subsequent ligation were designed and annealed followed by ligation into the digested vector. The sequences for the sgRNAs are listed in S1 Table. All plasmid sequences were verified using Sanger sequencing.

### Plasmid for monitoring GCPR activity

6xCRE mCherry plasmid in lentiviral backbone was cloned using lentiviral backbone plasmid pGH235 (gRNA-Zeo, from the Bassik lab) with the insert 6xCRE mCherry SV40 late Poly (A). Full plasmid sequences can be found in Supporting information.

### Isoproterenol treatment

Cells were seeded and left for 24 hours in regular media. They were starved with media with 0.1% FBS overnight, before the isoproterenol (Isoproterenol hydrochloride, Sigma 1351005-125MG) treatment with 1uM stimulation for 24 hours.

### Creation of stable cell line with AID*-dCas9

HEK293T and HEK293T GFP/mCherry were infected with virus for pGH389_AID*-dCas9-BlastR plasmid produced by Transomics with 10μg/mL polybrene. 3 days after infection, cells were selected with blasticidin 4ng/μL for at least 7 days.

### Single cell clones for HEK293T AID*-dCas9

In order to optimize the homogeneity of the cell population in the study, single cell clones were created for dCas9-AID* using Sony SH800 Cell Sorter. Clones were tested for Cas9 expression by Western Blot in Peggy Sue (Protein Simple). One million cells were lysed with RIPA buffer + protease inhibitors + DNAseI. The anti-Cas9 mouse antibody used was CRISPR CAS9 MAB 7A9 100UG (Epigentek), at 1:100. The anti-vinculin rabbit antibody used was EPR8185, Abcam ab129002, at 1:200. As secondary antibodies, we used Goat Anti-Mouse Secondary HRP Conjugate (ready-to-use reagent) (Protein Simple, Code 042–205) and Goat Anti-Rabbit Secondary HRP Conjugate (ready-to-use reagent) (Protein Simple, Code 042–206).

### Generation of KO cell lines for B2AR

HEK293T were transfected with Synthetic sgRNA AAGAAGGCGCTGCCGTTCCC (Synthego) and TrueCut Cas9 (RNP) (ThermoFisher), using Lipofectamine CRISPRMAX (ThermoFisher). Two days after transfection, genomic DNA was extracted to evaluate the knock-out efficiency for the pool of cells. The amplicon was amplified using oligos AACTCGCACCAGAAGTTGCC and GCACAGCACATCAATGGAAG, sequenced and analyzed using the ICE tool (Synthego). Single cell clones were plated with conditioned media and left to grow for 2 weeks. Genomic DNA was extracted from the single cell clones using QuickExtract DNA Extraction Solution (Lucigen). The same PCR as previously used was done again, using oligos AACTCGCACCAGAAGTTGCC and GCACAGCACATCAATGGAAG, sequenced and analyzed using the ICE tool (Synthego).

### Virus production for sgRNAs

Takara (Clonetech) Lenti-X Packaging Single Shots (4^th^ generation) were used following manufacture protocol. Supernatant was collected 48–72 hours post-transfection to infect cells (with 10μg/mL polybrene). For the timepoints experiment, when concentrated infection is indicated, Lenti-X concentrator (Clonetech) was used to concentrate the virus.

### sgRNA library production

sgRNAs were designed on the B2AR coding sequence along with an additional 50 base pairs at both ends of sequence using the CHOPCHOP tool [23–25] and the MIT tool [26]. Results were combined taking CHOPCHOP as reference and adding the 12 unique sgRNAs from MIT results. sgRNAs with off-targets with 3 or more mismatches and no BbsI targets (for cloning purposes) were kept. The final list contained 128 sgRNAs along the 1341bp of the B2AR coding sequence +/- 50bp (sgRNA list in S2 Table). To ensure diversity on the sgRNA infection, 9 million cells in a p10 plate were infected with the virus at low MOI (0.05) to ensure that at least 1000 cells were infected with the same sgRNA.

### Generation of mutants

2–3 days after transfection/infection/electroporation of sgRNA, sustained selection with puromycin 2ng/μL containing media was used for 14 days, unless shorter times are specified.

### siRNAs

siRNA ADRB2—assay ID s1122 (ref. 4427037), Silencer^™^ Select Negative Control No. 1 siRNA (ref. 4390843) from Thermo Fisher were used at 10 pmol/well in 24-well plate. ON-TARGETplus Human ADRB1 (153) siRNA–SMARTpool (ref L-005425-00-0005), ON-TARGETplus Non-targeting Control Pool (ref. D-001810-10-05) from Dharmacon were used at 50nM. RNA was extracted 72h after transfection using RNeasy Plus Mini Kit (Qiagen).

### RT-qPCR

Retrotranscription was performed with QuantiTect Rev. Transcription Kit (Qiagen) using 500ng of RNA. qPCR was performed with the Taqman assay (ThermoFisher). Taqman probes used were: Hs00240532_s1 ADRB2, Hs03003658_s1 ADRB2, Hs02330048_s1 ADRB1, Hs00265096_s1 ADRB1, Hs00609046_m1 ADRB3, Hs02786624_g1 GAPDH, Hs04420632_g1 GAPDH (ThermoFisher).

### Flow cytometry analysis and sorting

The Fortessa cytometer was used for analysis and a Sony SH800 Cell Sorter was used for sorting cells. The sample buffer was PBS-CMF + 10mM HEPES + 0.5% FBS and the Collection media growth media + 25mM HEPES.

### High content imaging

Red fluorescence was measured in a 96-well plate using IncuCyte Zoom (ThermoFisher Scientific).

For the library validation experiment in Fig 8, HEK-CRE-mCherry or HEK-CRE-mCherry B2AR-KO cells were transiently transfected with 50ng per well (in a 96 well plate) of plasmid with Lipofectamine 3000 reagents according to manufacturing protocols. 24 hours following transfection, titrating concentrations of isoproterenol were added to the transfection media and placed in IncuCyte instrument for real time monitoring of CRE-dependent mCherry activity.

### Genomic extraction and NGS

Genomic DNA was extracted from 0.5–2 million cells using the Quick-DNA micro prep kit (Zymo). The targeted loci were PCR amplified from 50 ng of genomic DNA using the primers shown in S3 Table with Kapa HiFi Hotstart ready mix (Kapa Biosystems). The PCR product was run for quality control in an Agilent TapeStation 4200. The concentration was measured using Qubit dsDNA HS Assay kit (ThermoFisher Scientific). Libraries were prepared using Taqmentation and the Nextera XT kit protocol (Illumina). Libraries were sequenced through NextSeq 500 (Illumina) with paired-end reads of length 76 bp.

### Bioinformatic analysis

BCL files from the sequencing were converted to FASTQ format using BCL2FASTQ (Illumina). SamTools was then used for alignment to the amplicon sequence using a quality score of 30. Mutagenesis rate per position in the amplicon was calculated as “reads of non-reference base allele/total reads”. Mutagenesis rate of 1 is the maximum and means all alleles have been edited. We normalized the mutagenesis values per sample obtaining mutagenesis z-scores per genomic position, and we computed the difference of z-scores between the mCherry negative sorted sample compared to the presorted sample. This variable appeared approximately normally distributed, so that we used the normal distribution function to computed p-values per genomic position per replicate, using as parameters its observed mean and standard deviation per replicate. We then used the Benjamini and Hochberg procedure for multiple test correction, obtaining adjusted p-values (i.e. q-values). We focused on those positions whose q-values were smaller than 5% in all replicates. Additionally, we computed which B2AR positions were targeted by sgRNA in our library, as follows. We considered a window of efficient mutation from -4 to +8 nucleotides from the 5’ mapping position of the sgRNA (called nucleotide +1). We considered all C or G nucleotides in such windows as targets for potential sgRNA-induced mutations to T or A, respectively. Thus, we considered B2AR protein positions as “targeted” if there was at least one potential sgRNA-induced mutation resulting in a missense substitution.

## Results

### Testing modified CRISPR-X at low MOI targeting GFP

**Fig 1:**
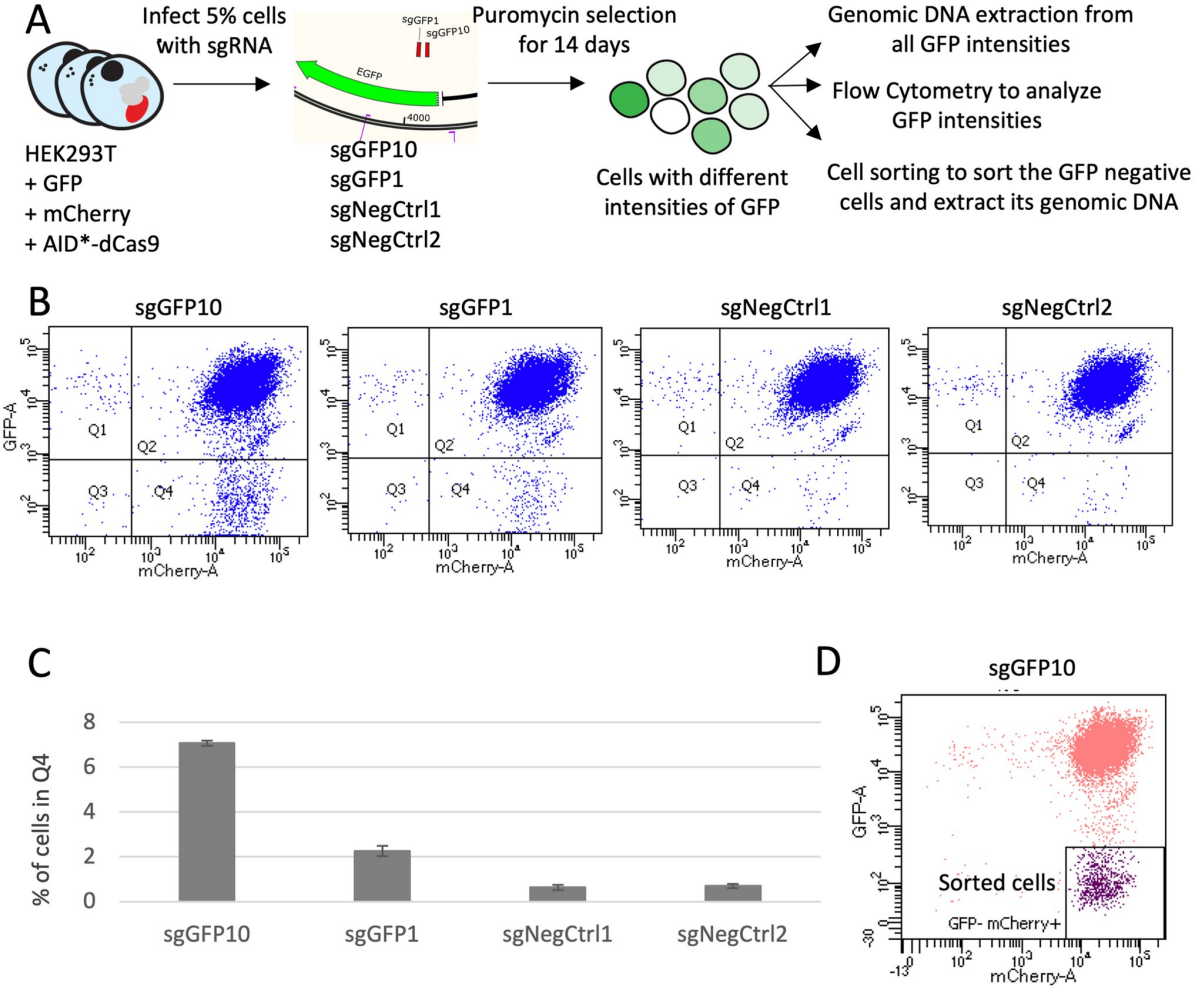
Testing modified CRISPR-X at low MOI targeting GFP. **A**. Design of the experiment. **B**. Flow Cytometry analysis plotting GFP and mCherry fluorescent intensities. **C**. The percentages of cells in Q4 quadrant for Fig 1B are graphed. The mean of three independent replicates with the standard deviation is shown. **D**. Flow cytometry analysis of sample sgGFP10, with sorted GFP-negative cells colored in purple.

For pooled screening, it is essential that each cell receives at most a single guide RNA (sgRNA), so it is crucial to use low multiplicity of infection (MOI) for plasmid delivery. Therefore, we first assessed whether low MOI could be successfully employed in our experimental framework. We employed the CRISPR-X base editing system for all our experiments [13], with minor variations compared to the original paper, as explained in Methods. CRISPR-X couples the inactive form of Cas9 (dCas9) to AID*Δ (Activation-induced cytidine deaminase) which converts cytidine to uracil. In our experiments to validate low MOI sgRNA delivery, we targeted a Green Fluorescent Protein (GFP) construct. We employed HEK293T cells with stable expression of GFP and of red fluorescent protein mCherry (a negative control not targeted by any sgRNAs). We employed two efficient GFP-targeting sgRNAs identified in Hess *et al*. (sgGFP10 and sgGFP1), and we additionally included two negative control sgRNAs (sgNegCtrl1 and sgNegCtrl2) (Fig 1A). Plasmids designed for sgRNA expression were packaged into a lentivirus and used to infect cells at 5% MOI. Infected cells were selected for 14 days with puromycin (selection marker in the sgRNA plasmid), then GFP levels were analyzed by flow cytometry. With sgGFP10, 7% of cells lost GFP fluorescence, indicating loss of function mutations (Fig 1B and 1C). With sgGFP1, 2% of the cells showed a diminished GFP signal. These results were in agreement with those obtained in [13] despite the different experimental systems (Hess *et al*. used K562 cells and an MS2 system). Next, cells were collected, and their genome analyzed by Next Generation Sequencing to assess mutagenesis. We sequenced unsorted cells for every sample, and we additionally analyzed sorted (GFP- mCherry+) cells for sgGFP10 (Fig 1D). While the flow cytometry analysis indicated the overall proportion of cells with loss of function mutations, the analysis of sequencing data allowed us to determine the precise mutation rate per position at the DNA level.

**Fig 2:**
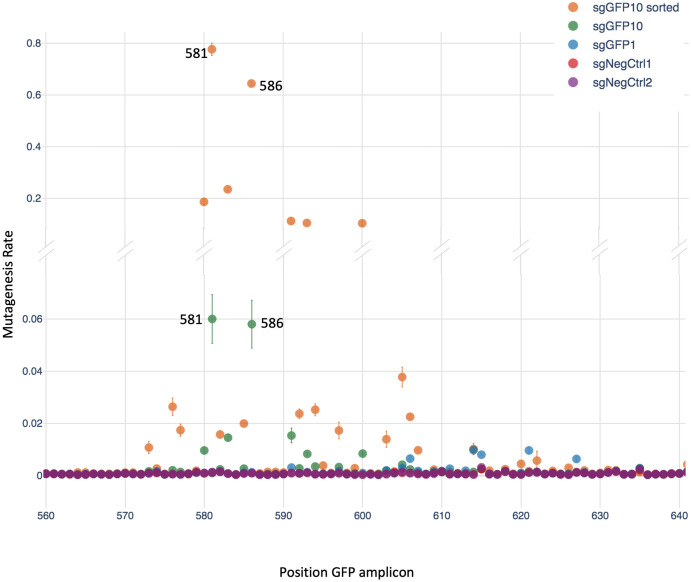
Mutagenesis rates with modified CRISPR-X at low MOI targeting GFP. Mutagenesis rate per position in the GFP region targeted with sgRNAs. The mean and standard deviation of three independent replicates are shown.

We found that the sgGFP10 sample had an overall higher mutation rate than sgGFP1 (Fig 2), consistent with its greater loss of fluorescence signal. The most highly mutated nucleotides were at positions 581 and 586, corresponding to residues 21 and 23 of GFP, both featuring about 6% mutation rate in the unsorted sgGFP10 sample. In the sgGFP10 sorted fraction (GFP negative), the same positions featured mutation rates of 77% and 64%, confirming the functional consequence of mutating these sites. Additionally, significant mutagenesis was seen at the neighboring residues. For the sgGFP1 unsorted sample, the mutagenesis rate per position was around 1% in the expected region, substantially higher than the background (<0.1%). These results showed that our experimental system could be successfully used at low MOI, so that we proceeded to apply it to study a GPCR protein.

### Targeting B2AR as a model GPCR

We decided to study GPCRs due to the considerable interest in this protein family in drug discovery. The β2 Adrenergic Receptor (B2AR) was chosen as a model GPCR as it has been extensively characterized. The MoA of isoproterenol was studied as it is an agonist molecule known to activate B2AR [27–29]. To enable a phenotypic readout of the base editing, it is essential to link the target protein activity to a fluorescent signal that can be employed to sort cells. Here, we monitored GPCR activity through an mCherry reporter whose promoter contained six copies of the cAMP response element (6xCRE-mCherry), so that it became expressed upon B2AR activation.

**Fig 3:**
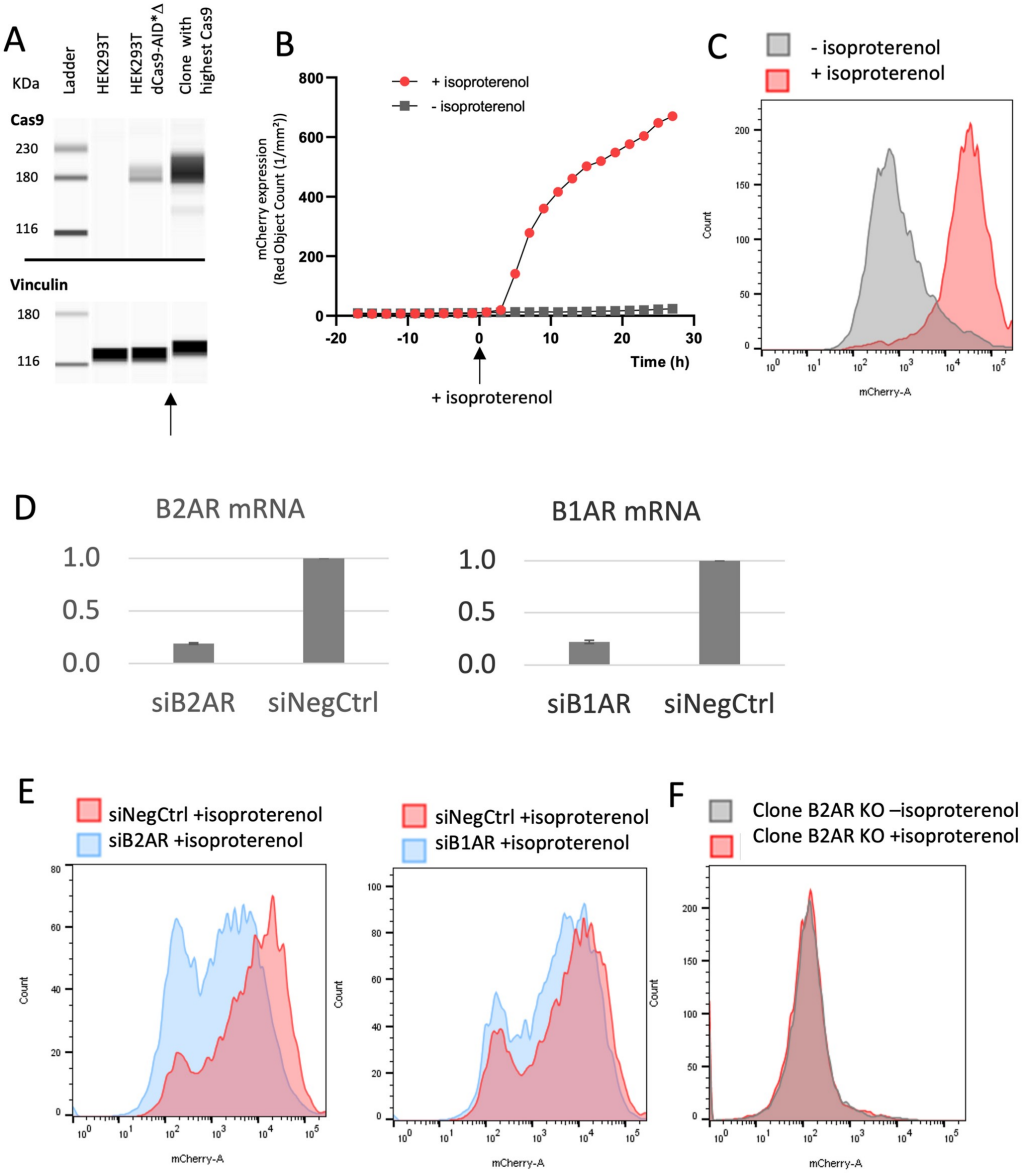
Targeting B2AR as a model GPCR. **A**. Western Blot for Cas9 expression levels in three cell lines: Wild-type HEK293T, HEK293T + dCas9-AID*Δ as a pool, and the single cell clone of HEK293T + dCas9-AID* selected for the highest expression of Cas9. Housekeeping protein vinculin is used as reference. Blots were cropped where indicated by the arrow. Full uncropped blots are provided in S1 Fig. **B**. Clone HEK293T dCas9-AID*Δ was infected with 6xCRE-mCherry reporter system and single cell clones were evaluated. The most homogeneous clone for mCherry expression was selected. mCherry red fluorescence was measured with high content imaging using a cell incubator imaging system. Cells treated with and without isoproterenol were monitored for 30 hours after stimulation. **C**. mCherry fluorescence intensity of the same single cell clone as panel B, 24 hours after isoproterenol treatment or in its absence. **D**. B2AR and B1AR mRNA expression 72h after transfection of 10pmol of siRNA. The mean of 4 replicates is shown. **E**, Left, mCherry fluorescence intensity of cells transfected with control siRNA or siRNA against B2AR. Right, mCherry fluorescence intensity of cells transfected with control siRNA or siRNA against B1AR. In both cases, cells were stimulated with isoproterenol after siRNA treatment. **F**. mCherry fluorescence intensity of knock-out cells for B2AR, with and without stimulation of isoproterenol.

Beforehand, we generated stable HEK293T cell lines with dCas9-AID*Δ by single cell cloning, selecting the clone with the highest levels of the Cas9 protein as probed by Western Blot (Fig 3A). The 6xCRE-mCherry reporter system was thus infected in HEK293T dCas9-AID*Δ cells, and single cell clones were grown. Next, we used flow cytometry to evaluate various clones and select one with an homogenous response to isoproterenol (i.e., a clone for which supplying isoproterenol resulted in a clear peak shift in fluorescent signal). Furthermore, mCherry fluorescence was evaluated over time using high content imaging (Fig 3B). This led us to choose a 24-hour isoproterenol treatment to ensure high expression of mCherry in further experiments. The intensity of mCherry fluorescence allowed the cell populations to be distinguished with and without isoproterenol stimulation, and therefore allowed sorting cells based on GPCR activity (Fig 3C).

Next, we evaluated the specificity of the 6xCRE reporter system for B2AR, since its homologs B3AR and B1AR might potentially also be activated by isoproterenol. HEK293T cells do not endogenously express B3AR and the expression of B1AR is very low [30]. We assessed B2AR and B1AR mRNA levels by RT-qPCR, after knocking them down using siRNAs. 72 hours after siRNA transfection, the mRNA level of B2AR was reduced to 19.2% and B1AR 22.1% of the normal expression (Fig 3D). Supporting specificity of the 6xCRE system for B2AR, the siRNA against B1AR did not downregulate the mCherry signal. In comparsion, the mCherry signal was reduced upon B2AR siRNA treatment, although we noted it was not eliminated completely (Fig 3E). We attributed this resilience to the natural signal amplification in the B2AR pathway, so that even a low level of B2AR expression results in full pathway activation. For a conclusive test of the specificity of 6xCRE reporter system, we also generated single cell clone knockouts (KO) for B2AR using a CRISPR-Cas9 system. Two different KO clones were tested, yielding the same result: after stimulation with isoproterenol, B2AR KO cells did not express any mCherry (Fig 3F).

Altogether, our results confirmed the specificity of the 6xCRE mCherry reporter system for B2AR activity. They also indicated that a nearly complete removal of B2AR may be necessary to suppress mCherry expression.

### Defining the optimal conditions for mutagenesis

**Fig 4:**
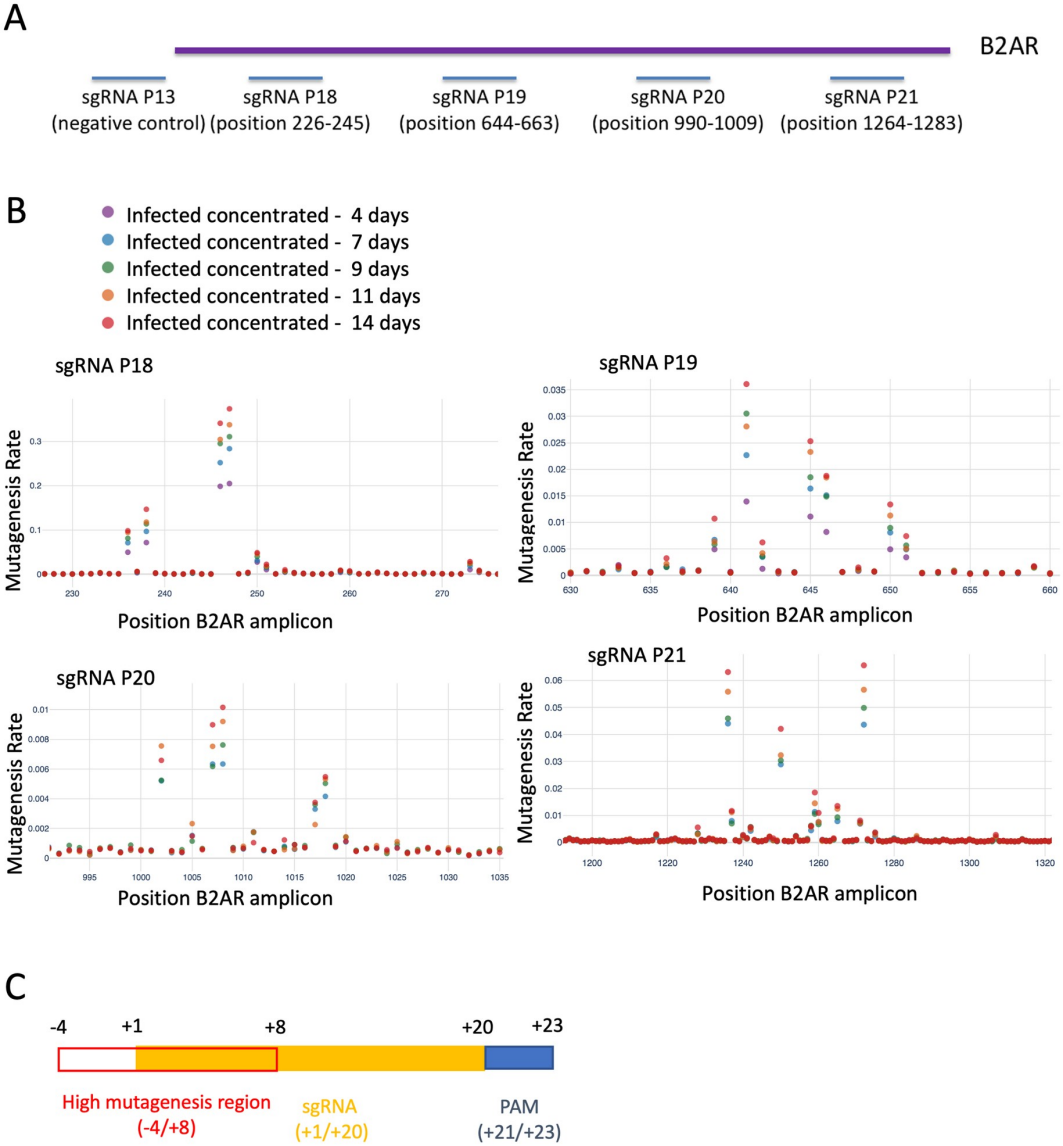
Defining the optimal mutagenesis conditions. **A**. Experiment design: 4 sgRNAs were designed to target regions of the B2AR gene (P18-P21). An untargeted negative control (P13) was also included. Positions on the B2AR amplicon are specified in the figure. **B**. Mutagenesis rate per B2AR position at different timepoints. For each sgRNA, the targeted and surrounding region in B2AR is shown. **C**. Diagram of a sgRNA. The region with increased mutagenesis rate, indicating high activity of the base editor, is highlighted in red.

We performed a series of experiments to determine the optimal conditions for mutagenesis, evaluating duration of treatment (timepoint), delivery method, and location of mutagenesis. In this phase, we employed four sgRNAs targeting B2AR along with a scrambled control (Fig 4A and S1 Table) which were delivered to HEK293T cells containing dCas9-AID*Δ and 6xCRE mCherry reporter (clonal). We delivered plasmids either via infection, concentrated infection (i.e., with a higher viral titer), transfection, or electroporation (see Methods). Genomic DNA was extracted at time points of 4, 7, 9, 11, and 14 days after puromycin selection, and sequenced by NGS.

**Fig 5:**
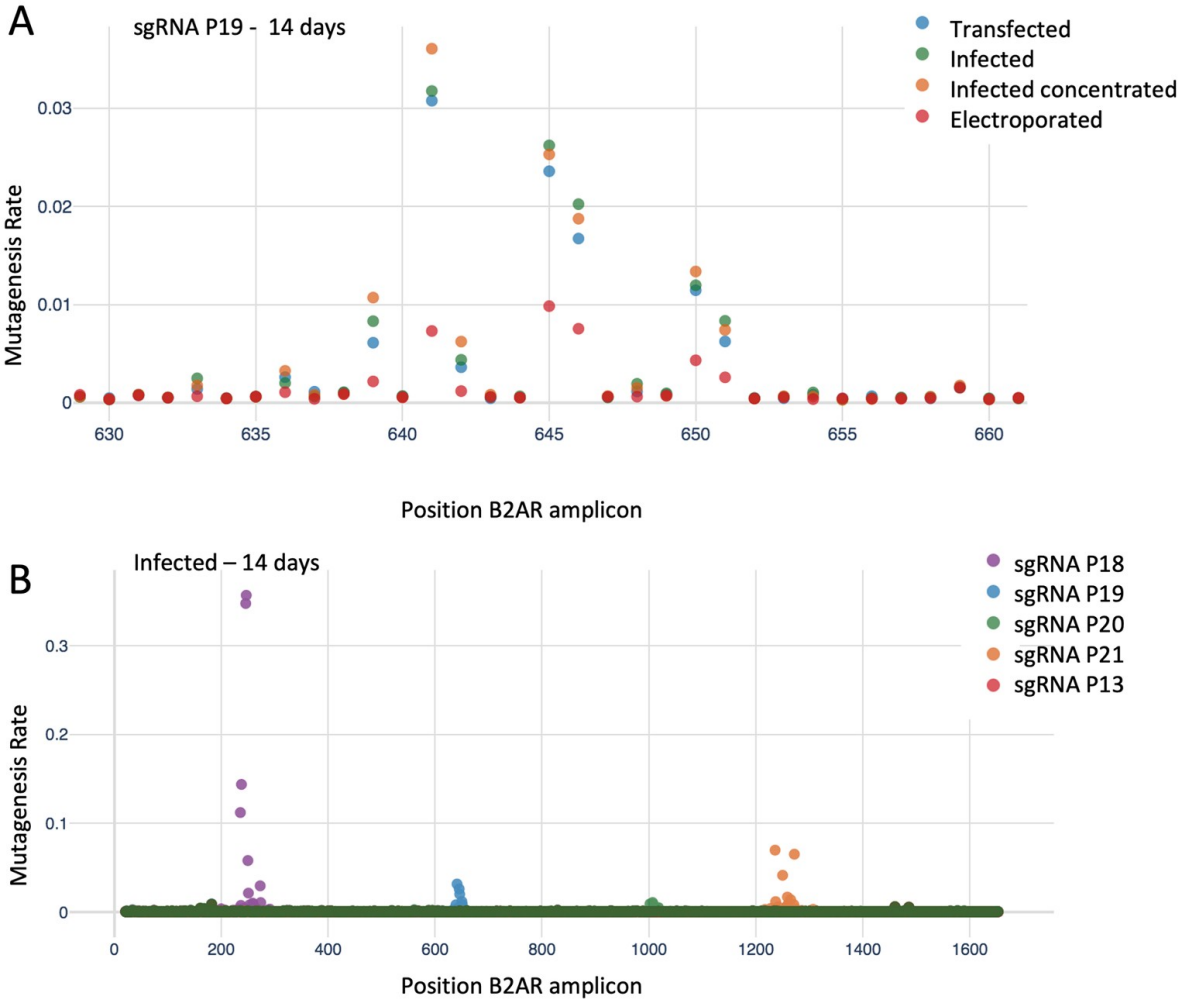
Defining the optimal mutagenesis conditions (continuation). **A**. Mutagenesis rate per position using different plasmid delivery methods. **B**. Mutagenesis rate per position for different sgRNAs.

Mutagenesis rates per sample and position are plotted in Fig 4B (time course), Fig 5 (different plasmid delivery methods and different sgRNAs), S2 and S3 Figs (combinations not shown in the main figures). We observed a consistent pattern of increasing mutagenesis over time, testifying to the slow time scale of the mutational process. Notably, the mutagenesis window along nucleotide positions for each sgRNA did not increase over time. No additional sites of mutation appeared at later timepoints; instead, we observed the same sites as early timepoints, but with increased mutagenesis. This allowed us to define a high mutagenesis region around each sgRNA. This region extended from position -4 to position +8 in respect to the 5’-end of the sequence matched by the sgRNA (Fig 4C). When comparing plasmid delivery methods, similar efficiencies were seen for infection and transfection, while electroporation showed decreased mutagenesis rates (Fig 5A and S3A Fig). Finally, as expected, we noted that different sgRNAs featured a wide range of mutagenesis rates (Fig 5B and S3B Fig), ranging from 1% to more than 30%.

### Pooled screening for B2AR

**Fig 6:**
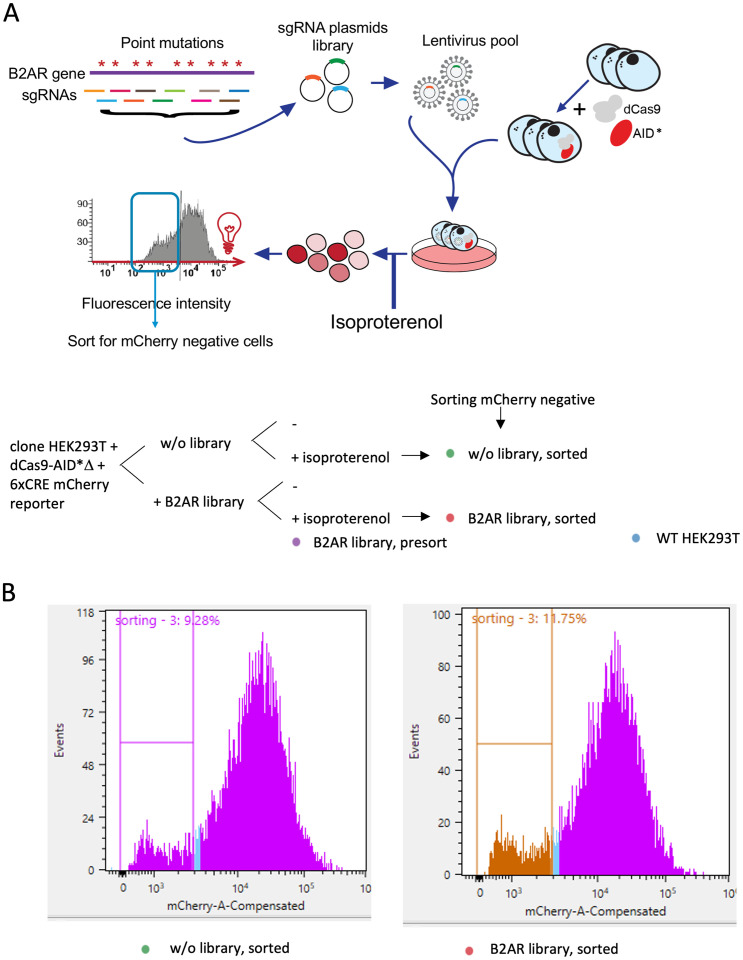
Pooled screening for B2AR. **A**. Design of the experiment. The sgRNA library containing 128 sgRNAs was infected into clone HEK293T + dCas9-AID*Δ + 6xCRE mCherry reporter at about 0.05 MOI. Cells were selected with puromycin, and treated with isoproterenol to stimulate B2AR activity. Cell pellets were obtained for genomic extraction before sorting (presort cells) and sorted for the mCherry negative in both populations (the infected with the B2AR library and the non-infected cells). WT HEK293T cells do not contain the CRISPR-X system, and are used here as negative control. **B**. mCherry fluorescence intensity histogram, showing the cut-off for sorting mCherry negative cells.

In an attempt to uncover residues of B2AR that are important in isoproterenol-mediated activation, we proceeded to perform a pooled screening (Fig 6A). An sgRNA library was designed by tiling along the gene, using two separate algorithms to design sgRNAs on the B2AR coding sequence extended by 50 base pairs at both ends. The CHOPCHOP tool [23–25] and MIT CRISPR tool [26] yielded 192 and 203 candidate sgRNA sequences, respectively. The results were combined taking the CHOPCHOP sequences as the reference and then adding the additional 12 unique sgRNAs from MIT. sgRNAs were evaluated for off-target matches and retained only those with no less than 3 mismatches with any genomic site other than B2AR (and without the BbsI restriction enzyme site, for cloning reasons). The final design consisted of 128 sgRNAs along the 1341 base-pairs of the B2AR gene.

The sgRNA library was infected into the HEK293T-dCas9-AID*Δ/6xCRE mCherry reporter at 5% MOI. Three days after infection, cells were selected using puromycin for 14 days. On the last day, cells were treated with isoproterenol to stimulate B2AR activity. We then sorted cells by flow cytometry, selecting the mCherry-negative population (i.e., cells not responding to isoproterenol). Cell pellets were obtained for genomic DNA extraction both before sorting (presort cells) and after (sorted cells), and non-infected cells were analyzed as negative control (Fig 6A). The proportion of mCherry negative cells was about 3% higher in samples with the sgRNA library than those without (Fig 6B), showing a small but consistent effect in all of our 3 replicates.

**Fig 7:**
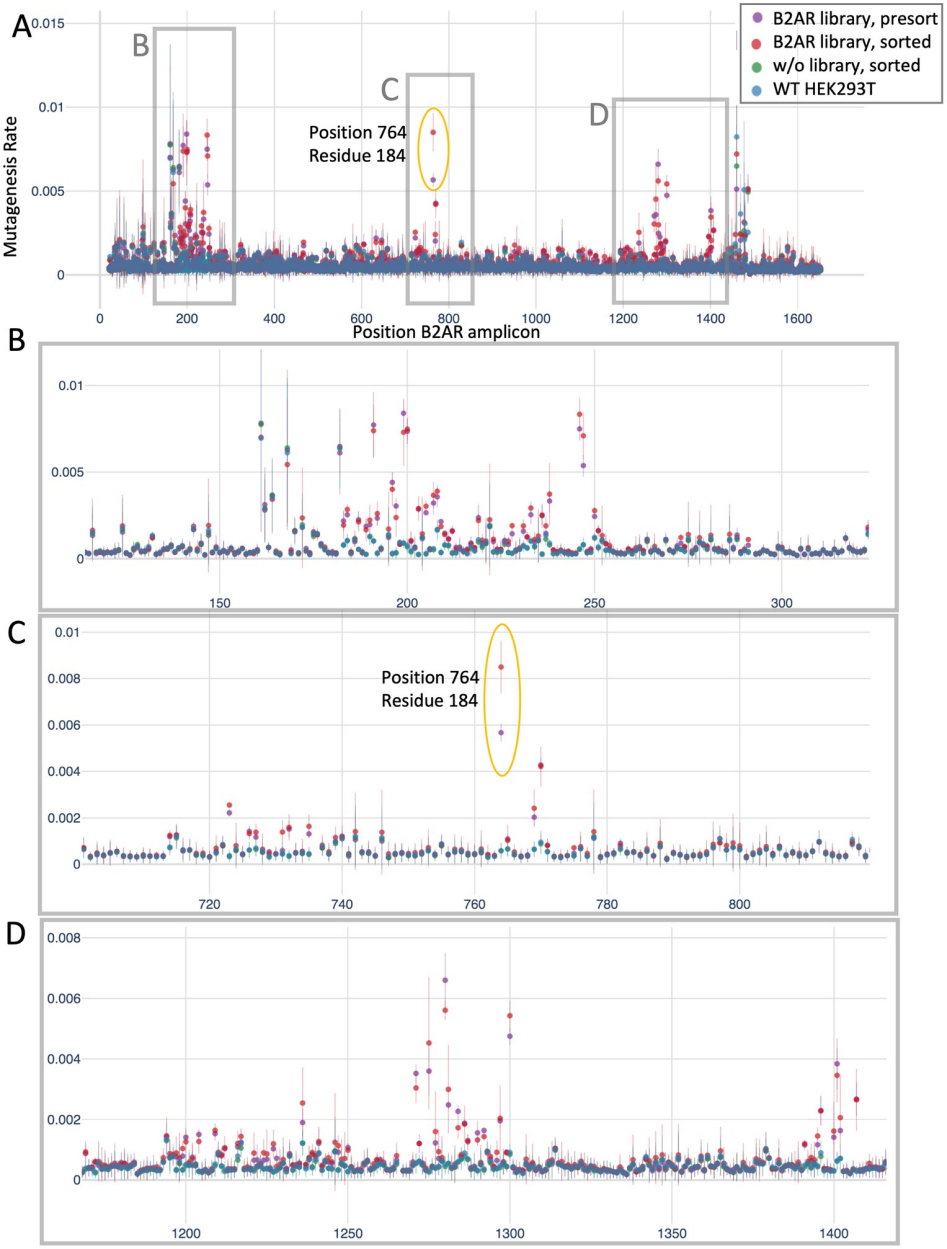
Mutagenesis rates for pooled screening for B2AR. **A**. Mutagenesis rate per position of the B2AR amplicon. The mean and standard deviation of three independent replicates are shown. **B, C, D**. Zoom-ins of the B2AR regions marked in panel A.

We next analyzed sequencing data to assess mutagenesis. More than 95% of the mutations occurred at G and C bases, as expected for the CRISPR-X base editor. We observed overall low mutation rates (less than 1% for all positions, as shown in Fig 7A). This was expected given that the library contained 128 sgRNAs, and the mutagenesis at any particular position was diluted by the library diversity (e.g. for a library of 100 sgRNAs, only 1/100 of the cells contain a particular sgRNA). Next, we searched for nucleotide positions with higher mutagenesis rate in sorted than presort cells, pinpointing the functional sites of interest (see Methods). After normalizing mutagenesis rates per sample, we computed the difference between the sorted and presort samples, which was approximately normally distributed (S4A Fig). After filtering, we detected a single hit with significant q-values in all three replicates (S4B Fig): a mutation at B2AR position 764, corresponding to residue 184 (Fig 7C), which converts a cysteine to tyrosine (C184Y). This cysteine residue is known to form a key disulfide bond with residue 190, helping to form and stabilize the ligand binding site [31]. No other position passed our selection criteria, as the mutational rate differences between the presort and sorted cells were small or inconsistent (Fig 7B and 7D and S4B Fig).

### B2AR with C184Y mutation is less sensitive to isoproterenol stimulation

**Fig 8:**
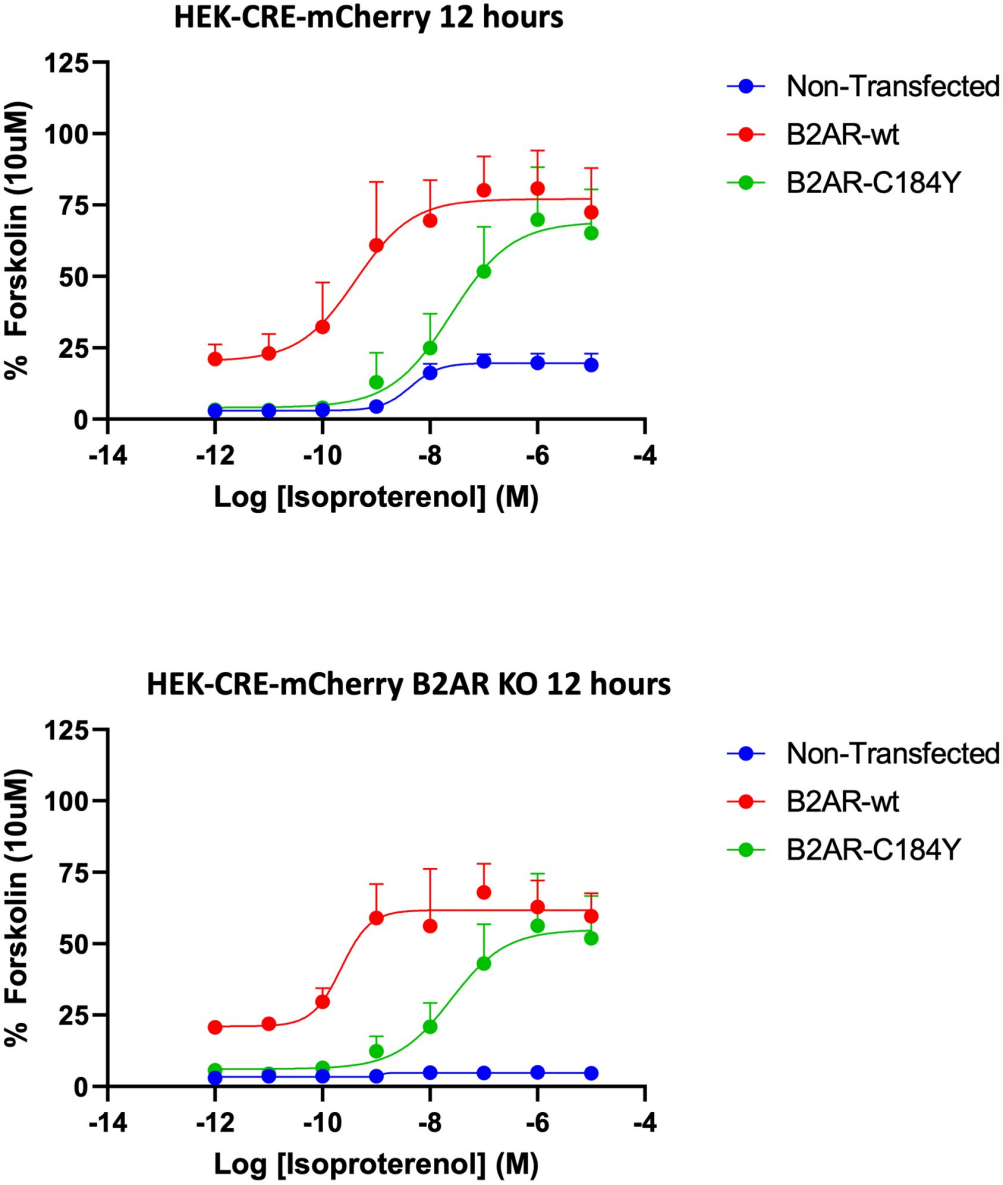
The C184Y mutation reduces B2AR sensitivity to isoproterenol. Reporter activity to titrating concentrations of isoproterenol of cells with or without endogenous B2AR (HEK-CRE-mCherry or HEK-CRE-mCherry B2AR-KO, respectively) after transient transfection of B2AR-wt or B2AR-C184Y. CRE-mCherry activity was assayed 12 hours after isoproterenol stimulation (other time points are shown in S5 Fig). Isoproterenol dependent CRE-mCherry activity is expressed as a percentage of 10μM forskolin (used as internal positive control, as it activates cAMP downstream of B2AR) response. Results are plotted of mean data of N = 3 ± SEM.

Next, we proceeded to validate the residue returned by our pooled screening. We overexpressed separately WT B2AR and B2AR with the C184Y mutation in either wild-type or B2AR knock-out cells. We thus monitored CRE-mCherry activity over time after stimulation, testing a wide range of isoproterenol concentrations. The complete response curves at 10, 12, and 14 hours are illustrated in S5 Fig, and calculated pharmacological parameters are shown in S4 Table. The comparison of samples at a representative time point (12 hours) is shown in Fig 8.

Strikingly, cells with endogenous B2AR (HEK-CRE-mCherry) transfected with wildtype B2AR were far more responsive to isoproterenol than those transfected with B2AR-C184Y. Indeed, the negative logarithm of isoproterenol concentration required to reach half of the maximum mCherry signal (p[A]_50_) was 9.03 ±0.6 for the former and 7.59 ±0.4 for the latter (Table 1), indicating a ~30-fold difference in sensitivity. In HEK cells without any endogenous B2AR (HEK-CRE-mCherry B2AR-KO), the difference was even more pronounced, ~55-fold. These results show that the C184Y mutation makes B2AR less sensitive to isoproterenol stimulation, validating its discovery in our screening.

**Table 1:** Effect of B2AR-C184Y mutation on isoproterenol dependent CRE-mCherry activity.

Cell Background	Plasmid	N	p[A]_50_	E_max_	α
HEK-CRE-mCherry	Non-transfected	3	8.29±0.3	19.65±5.5	0.25
	B2AR-wt	3	9.03±0.6	79.06±22.8	1.0
	B2AR-C184Y	3	7.59±0.4	68.70±29.7	0.87
HEK-CRE-mCherry B2AR-KO	Non-transfected	3	N/A	3.23±0.3	0.05
	B2AR-wt	3	9.28±0.4	64.64±16.7	1.0
	B2AR-C184Y	3	7.55±0.4	55.00±28.5	0.85

Parameters are derived from Fig 8 using isoproterenol in B2AR-wt transfected cells in HEK-CRE-mCherry or HEK-CRE-mCherry B2AR-KO as reference activity and assigned value of 1. p[A]_50_ is the concentration of agonist that gives 50% of the maximum measured response. Emax is the calculated maximum response of the agonist. α is the ratio between the standard condition (in this case B2AR-wt plasmid)/variant’s Emax.

### Positional analysis of B2AR

Our results indicated that our screening was successful in identifying a bona-fide functional residue. Nevertheless, we were surprised that our procedure had returned a single hit, as we were expecting multiple such functional positions. Therefore, we went back to the B2AR sequence and, for each position, we traced where it had been lost or filtered out in our analysis. First, we considered whether every given amino acid was “targeted” in our experiments, i.e., if a missense mutation could be introduced at this position by any sgRNA included in our library (see Methods).

As shown in S6 Fig, the majority of B2AR amino acids (63%) were not targeted, either because no missense mutation could be introduced by the CRISPR-X base editor (as it can edit only C/G positions), or because no sgRNA could be designed at suitable location. Another 30% of B2AR sequence was targeted but it was not observed mutated in our samples, presumably due to inefficient mutagenesis or low sgRNA representation. Therefore, we estimated that we effectively interrogated 6.5% of the protein sequence, ultimately yielding one amino acid (i.e., 0.2% of sequence) as positive hit, which we successfully validated. We concluded that our procedure was successful in assaying functional sites, but it profiled a rather narrow set of residues. Next, we discuss the technical limitations that hindered functional identification at larger scale, which will be instrumental for further applications of this approach.

## Discussion

The objective of this study was to evaluate the utility of base editors in early drug discovery, as a tool for understanding mechanisms of compound pharmacology and target biology. This has been traditionally done through alanine scanning and site directed mutagenesis, however the development of CRISPR-based gene editing has created the opportunity for a less time-consuming and more high-throughput approach. After designing an effective reporter system, the delivery a pool of sgRNA at a low MOI followed by flow cytometry allows the separation of cells into responding and non-responding cohorts, and analysis of sequencing data pinpoints to those mutations with functional effects.

Here, we demonstrated the feasibility of low MOI infection for pooled editing, and we optimized the experimental conditions. Our results indicated that modified CRISPR-X is effective when sgRNA delivery is performed by transfection or infection, and puromycin selection is performed for 14 days, resulting in high mutagenesis at position -4/+8 from the sgRNA (with mutation rates ranging between 1% and 30% in non-pooled experiments). Nevertheless, our results show that limitations related to the base editor system hindered a majority of the B2AR sequence being effectively interrogated in our screening. This was because the specific mutagenesis type did not allow editing certain codons; no suitable sgRNAs could be designed in some locations; and the efficiency of mutagenesis was insufficient in some positions.

The type and efficiency of mutagenesis are essential factors to consider for the success of this approach. Efficiency may be increased by exploring modifications to the experimental set up. One option is introducing multiple copies of the base editor, either beforehand while creating the cell line, within the sgRNA plasmid, or by co-transfection. Also, adopting more recently developed base editors such as evoCDA1-BE4max, with reported mutagenesis efficiency reaching up to 80%, may improve the outcome of this type of experiment [32].

A higher mutagenesis rate can also mitigate another potential problem, which may plausibly have been detrimental to our study: the overall ploidy (number of complete sets of chromosomes) of the cell line used. In case of haploid cells, a single mutation effectively alters all protein products for the target gene. In diploid cells, however, some amount of wild-type products will be synthesized unless both alleles are mutated. Complete allelic mutation is a feasible scenario only if mutagenesis rates are sufficiently high. The HEK293T cells used here were verified to be triploid (FISH showed 91% of the cells are triploid), which diluted mutagenesis over 3 alleles. The cell line is an essential choice for this approach: if available, cells with fewer alleles as possible for the target gene should be used.

All experiments were performed in three independent replicates. This revealed to be essential to obtain genuine results. In fact, samples featured highly variable mutagenesis across positions: single-sample analysis would have resulted in many potential hits even after multiple test correction, which were not validated across replicates (S4B Fig). Using consistency among replicates as filtering criteria, a single, yet solid, screening hit was obtained, which withstood the subsequent rigorous experimental validation. Our procedure was ultimately successful, but, in hindsight, we recommend to assess the statistical power of the screening in detail beforehand.

Finally, we believe that the most important factor in pooled screenings is the choice of the reporter system. One important characteristic is the specificity of signal for the target gene, which was well validated in our case. Another aspect, which proved problematic in our work, is the resolution of response between functional and disrupted phenotypes, essential for the accurate sub-setting of populations by flow cytometry. While the 6xCRE mCherry reporter system has more than sufficient resolution for traditional applications, its breadth of response makes it difficult to fully differentiate the subtle effects studied here, since cell populations have overlapping tails in their fluorescence signals. Much of this may be due to some of the cells still having at least one wild-type copy of the allele. Indeed, our siRNA experiments showed that only 19% of the mRNA level was sufficient for a substantial activation of the B2AR pathway, so that a single remaining non-mutated allele is expected to be sufficient for activation. This likely allowed cascade amplification as is typical of many signaling receptor pathways. An assay designed to monitor a more upstream element of the pathway could help reduce the problems of amplification, but may require a tradeoff for sensitivity.

**Fig 9:**
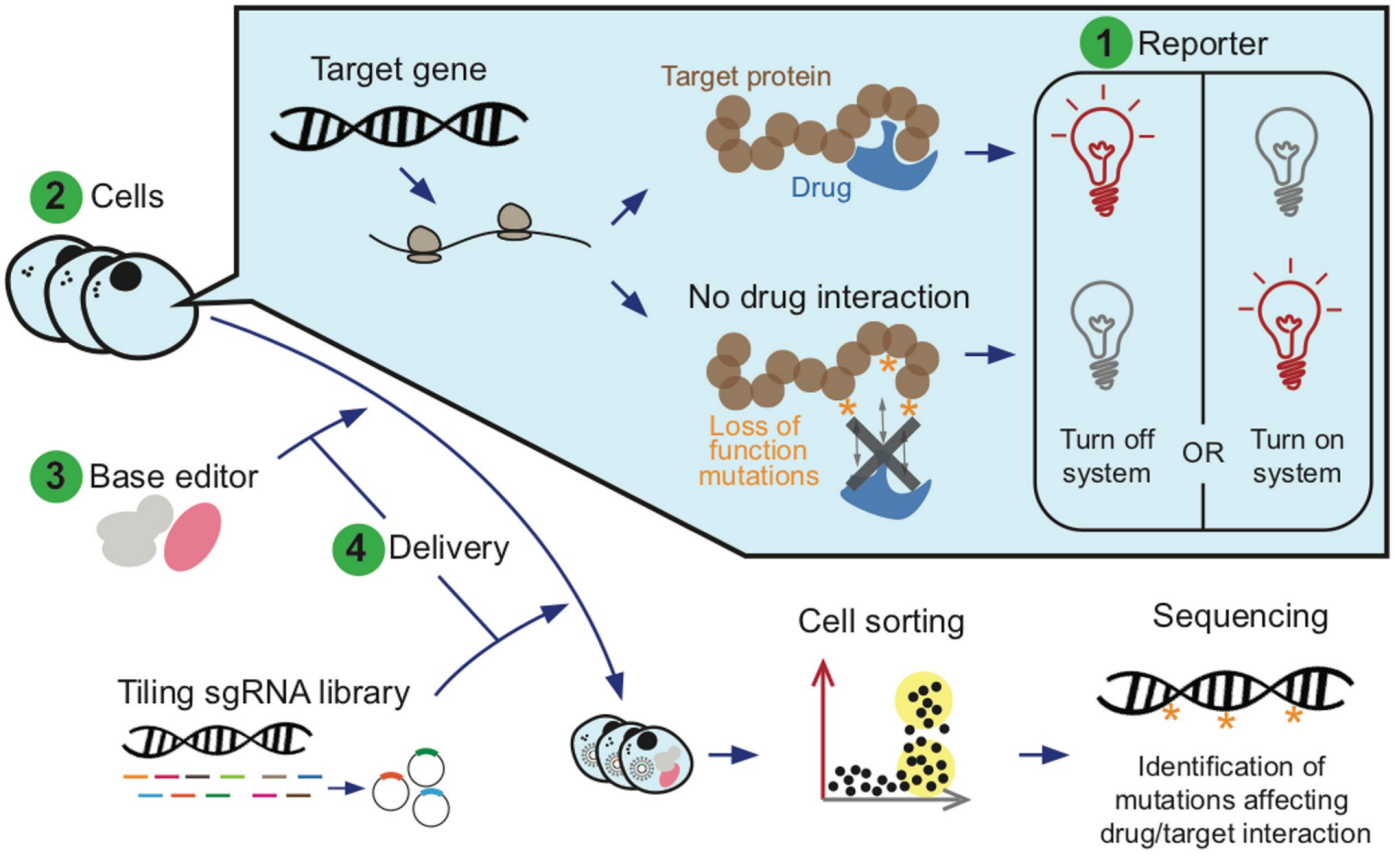
Graphical conclusions. Roadmap for future studies of this kind, defining the most important points to consider during study design, and where to spend effort in optimization.

Our design consisted of screening for loss-of-function mutations with a turn-off reporter system (i.e., wherein signal disappears upon occurrence of functional mutations) (Fig 9). There are alternative designs that may obviate some of the limitations we presented. A key point is that functional mutations should ideally have a *dominant effect* on the fluorescent signal. This may be obtained by employing a turn-on system, where signal is activated when the interaction between drug and target is disrupted by mutations. This framework may greatly facilitate meaningful sorting and thus functional identification, although designing and optimizing such a system is typically more challenging. To investigate B2AR, we performed early experiments to set up a turn-on system by employing a negative allosteric modulator (compound-15 [33]) together with isoproterenol (agonist) aiming to identify mutations which hindered compound-15’s effect. We also tried some weak/partial agonists for B2AR, such as salbutamol, dopamine, alprenolol and 1,2-dihydroxybenzene. Yet, we saw that the effects obtained were too weak to report significant differences in the 6xCRE mCherry system. Despite the impracticability of this particular system, we strongly recommend to preferentially seek a turn-on strategy for pooled editing MoA studies.

## Conclusions

Base editing technology has a great potential to quickly help decipher the mechanism of action of multiple drugs. Here, we implemented a modified CRISPR-X based pool editing system for MoA studies, and we applied it to study the B2AR agonist isoproterenol. We successfully conducted a proof-of-principle use of this system, but various limitations restricted its discovery power. Thus, we build on this experience to outline a “roadmap” for future studies of this kind, defining the most important points to consider during study design, and where to spend effort in optimization (see Fig 9):

1. After choosing the target gene and drug to study, the most crucial factor is the design of the **reporter**. Most compelling, its signal must clearly differentiate the active and inactive states of drug/target interaction. Moreover, the system should be activated uniquely by the protein of interest. Regardless of whether one seeks to find loss-of-function or gain-of-function mutations, it is recommended to set up a turn-on system, in which functional mutations activate (rather than deactivate) fluorescence. Finally, we recommend optimizing the signal resolution of drug response.
2. Second, the decision of which **cell line** to use is essential. This must be chosen in first place to be a suitable system to study the target pathway. Additionally, the ploidy for the target gene should be considered, preferentially selecting cells with fewer sets of alleles. Ideally, a haploid state would facilitate the identification of functional recessive mutations.
3. Third, one must pick which **base editor** system to employ. The most important feature is the efficiency of mutagenesis: the discovery power of the screening is largely dependent on it, so that it is worth spending effort to optimize it as much as possible. The types of the mutations that the editor can introduce must also be considered, as it determines which sites can be targeted and assayed functionally. When comparing different options, one should evaluate which fraction of coding sequence can be interrogated through non-synonymous mutations.
4. Fourth, one must decide which **delivery** method to use, both for the sgRNA library and for the base editor components. Ideally, available options should be tested during project development, optimizing the signal response to drug and the mutagenesis rate.
5. Lastly, **discovery power** must be assessed and boosted if necessary. On the one hand, it is important to plan ahead the final statistical analysis as much as possible. On the other hand, including several experimental replicates is essential to obtain results that are robust and reproducible, since individual samples may feature high levels of noise.

Base editing technologies show great promise to decipher the MoA of drugs. Here, we explored optimal conditions of application, and we defined driving principles that will facilitate future studies of this type.

## Supporting information

S1 Fig Uncropped blot corresponding to Western Blot from Fig 3A. Lanes marked in blue below are the ones of Fig 3A. Clone 10 is the single cell clone selected with the highest expression of Cas9.
(TIF)

S2 Fig A. Mutagenesis rate per position for B2AR amplicon showing different timepoints, per sgRNA, for infected samples. B. Mutagenesis rate per position for B2AR amplicon showing different timepoints, per sgRNA, for transfected samples. C. Mutagenesis rate per position for B2AR amplicon showing different timepoints, per sgRNA, for electroporated samples.
(TIF)

S3 Fig A. Mutagenesis rate per position for B2AR amplicon showing different delivery methods of the sgRNA. B. Mutagenesis rate per position for B2AR amplicon showing different sgRNA efficiencies.
(TIF)

S4 Fig A. Distribution of the difference between the normalized mutation rates of sorted and presort samples. The mean and standard deviations of each replicate are written within the plot. B. Scatterplots showing the q-values corresponding to each B2AR position, compared across pairs of replicates (see axis labels). Note that the values displayed are log10(q-value). The single position reaching significance in all replicates is colored and labelled in red (C184Y mutation).
(TIF)

S5 Fig Separate time point data for isoproterenol dependent CRE-mCherry response. Over three time points, 10, 12, and 14 hours, post isoproterenol stimulation. A predicted, time-dependent accumulation of mCherry intensity in both reporter cell backgrounds was observed for all conditions. Isoproterenol dependent CRE-mCherry activity is expressed as a percentage of 10μM forskolin, results are plotted of mean data of N = 3 ± SEM. Data was normalized to the 14h timepoint.
(TIF)

S6 Fig The plot shows the outcome of our screening procedure, traced back for each B2AR amino acid (Methods). Some positions could not be targeted in the first place (blue), meaning that there were no sgRNA in the library predicted to introduce missense mutations at these codons. Others were targeted but never observed mutated (green; z-score<2 in all samples with the sgRNA library). The rest of positions were effectively interrogated by our screening, and resulted either negative (yellow) or positive hits (red; i.e., mutations reducing the B2AR response to isoproterenol).
(TIF)

S1 Table sgRNA sequences. (DOCX)

S2 Table B2AR library sgRNAs. Sequences of the 128 sgRNAs used for the pooled library against B2AR (in supplementary Excel file “S2 Table_B2AR library sgRNAs.xlsx”).
(XLSX)

S3 Table Oligos to PCR genomic DNA. (DOCX)

S4 Table Effect of B2AR-C184Y mutation on isoproterenol dependent CRE-mCherry activity over time. Parameters are derived from S5 Fig.
(DOCX)

S1 Plasmid map sgRNA plasmid map: P5_pGH234_pMCB424 (GH020 with Ef1-puro). (DNA)

S2 Plasmid map Base editor (dCas9-AID*Δ) plasmid map: P10_pGH389_pMCB592 (AIDdelta_Mut7_3-dCas9-Blast). (DNA)

S3 Plasmid map 6xCRE mCherry reporter plasmid map: P17_6xCRE mCherry lenti zeo. (DNA)

S1 Raw images (PDF)

## Abbreviations

B2AR: β2 Adrenergic Receptor
Cas9: CRISPR Associated Protein 9
CRISPR: Clustered Regularly Interspaced Short Palindromic Repeats
DNA: Deoxyribonucleic Acid
GFP: Green Fluorescent Protein
GPCR: G-protein coupled receptor
MoA: Mechanism of Action
MOI: Multiplicity of Infection
sgRNA: single guide Ribonucleic Acid
