# Supplementary figures and images for: Roadmap for the use of base editors to decipher drug mechanism of action
Source: PLoS One. 2021 Sep 21;16(9):e0257537. doi: 10.1371/journal.pone.0257537 (PMC8454938; doi:10.1371/journal.pone.0257537)

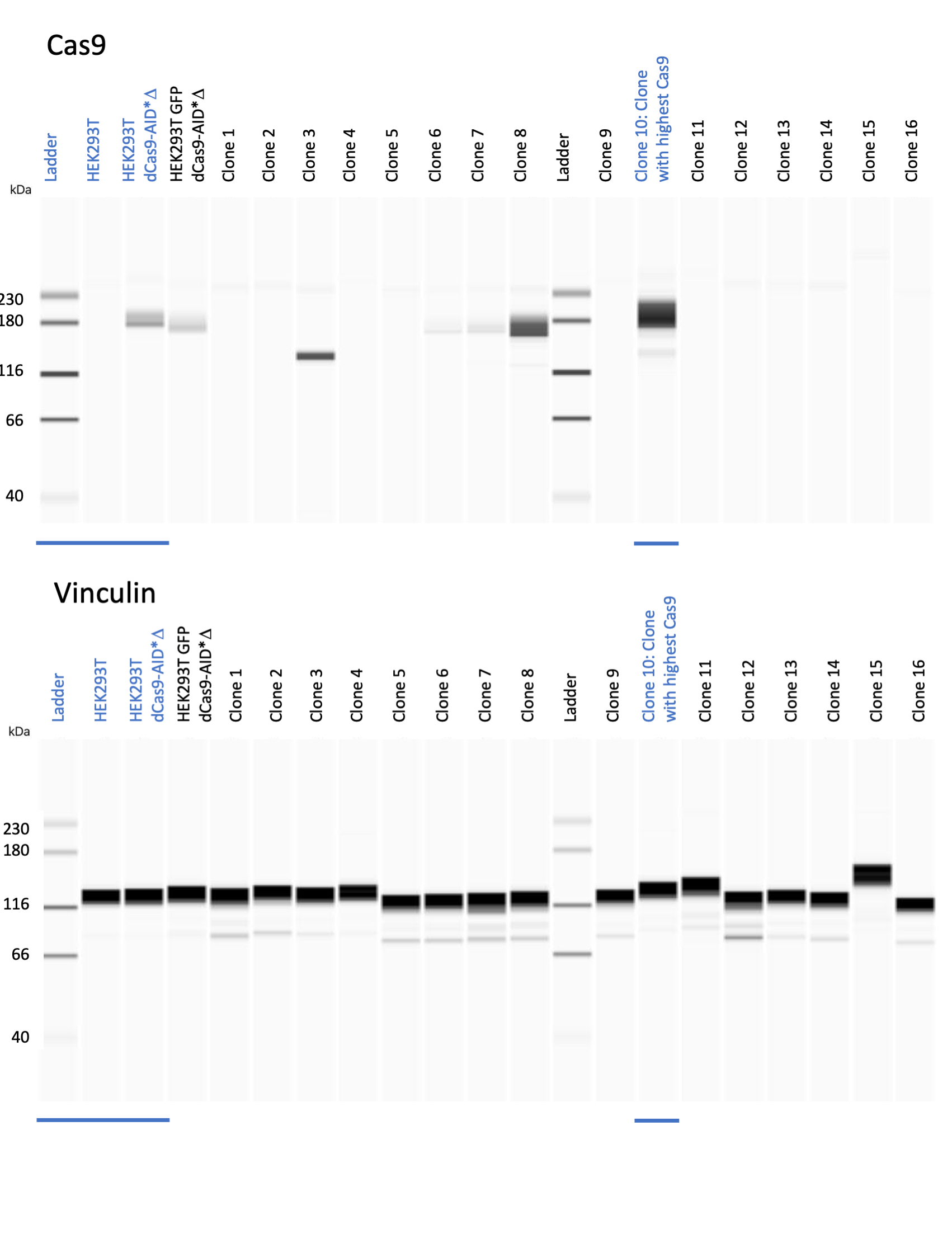

Supplement: S1 Fig — Lanes marked in blue below are the ones of Fig 3A. Clone 10 is the single cell clone selected with the highest expression of Cas9. (TIF) [file pone.0257537.s001.tif]

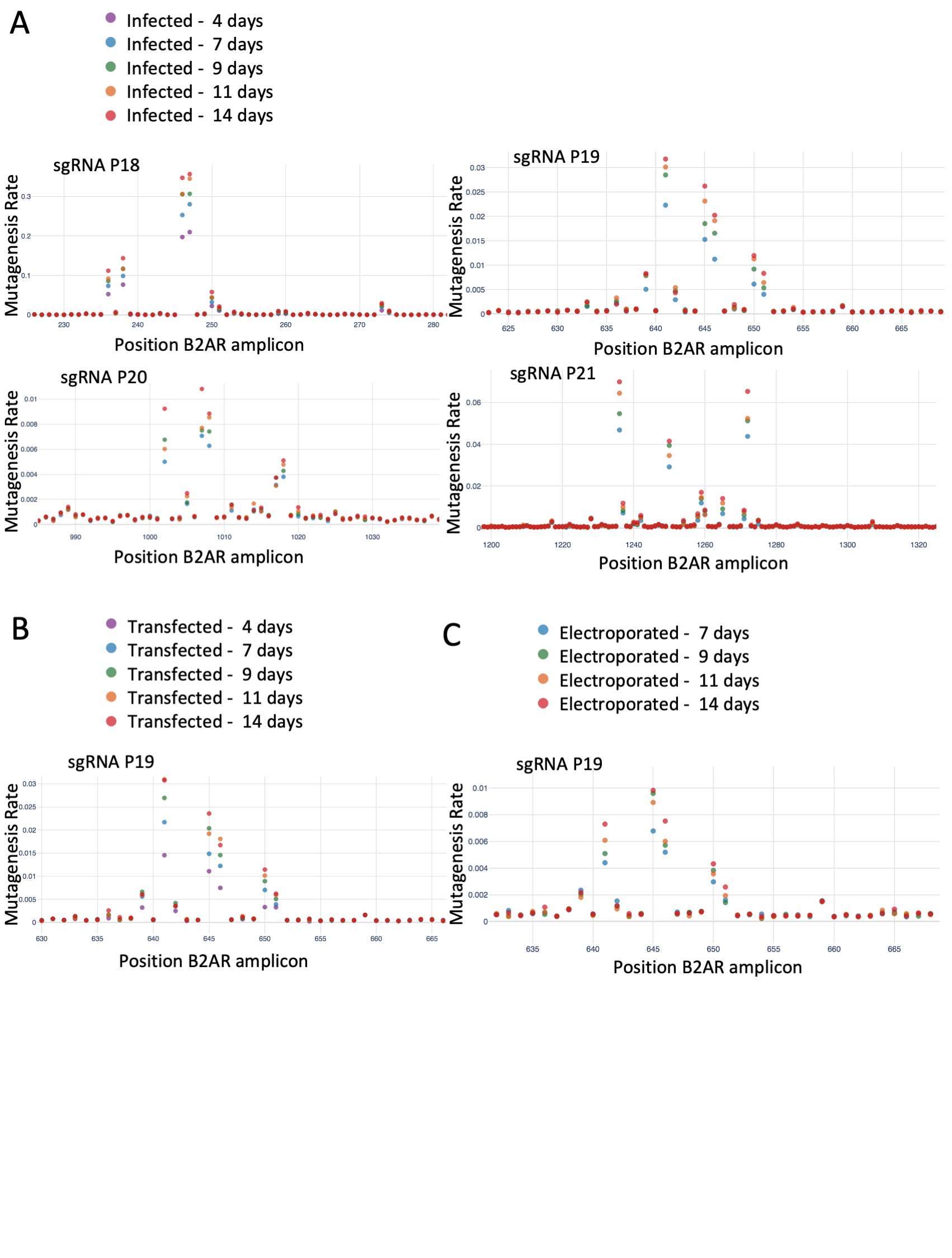

Supplement: S2 Fig — A. Mutagenesis rate per position for B2AR amplicon showing different timepoints, per sgRNA, for infected samples. B. Mutagenesis rate per position for B2AR amplicon showing different timepoints, per sgRNA, for transfected samples. C. Mutagenesis rate per position for B2AR amplicon showing different timepoints, per sgRNA, for electroporated samples. (TIF) [file pone.0257537.s002.tif]

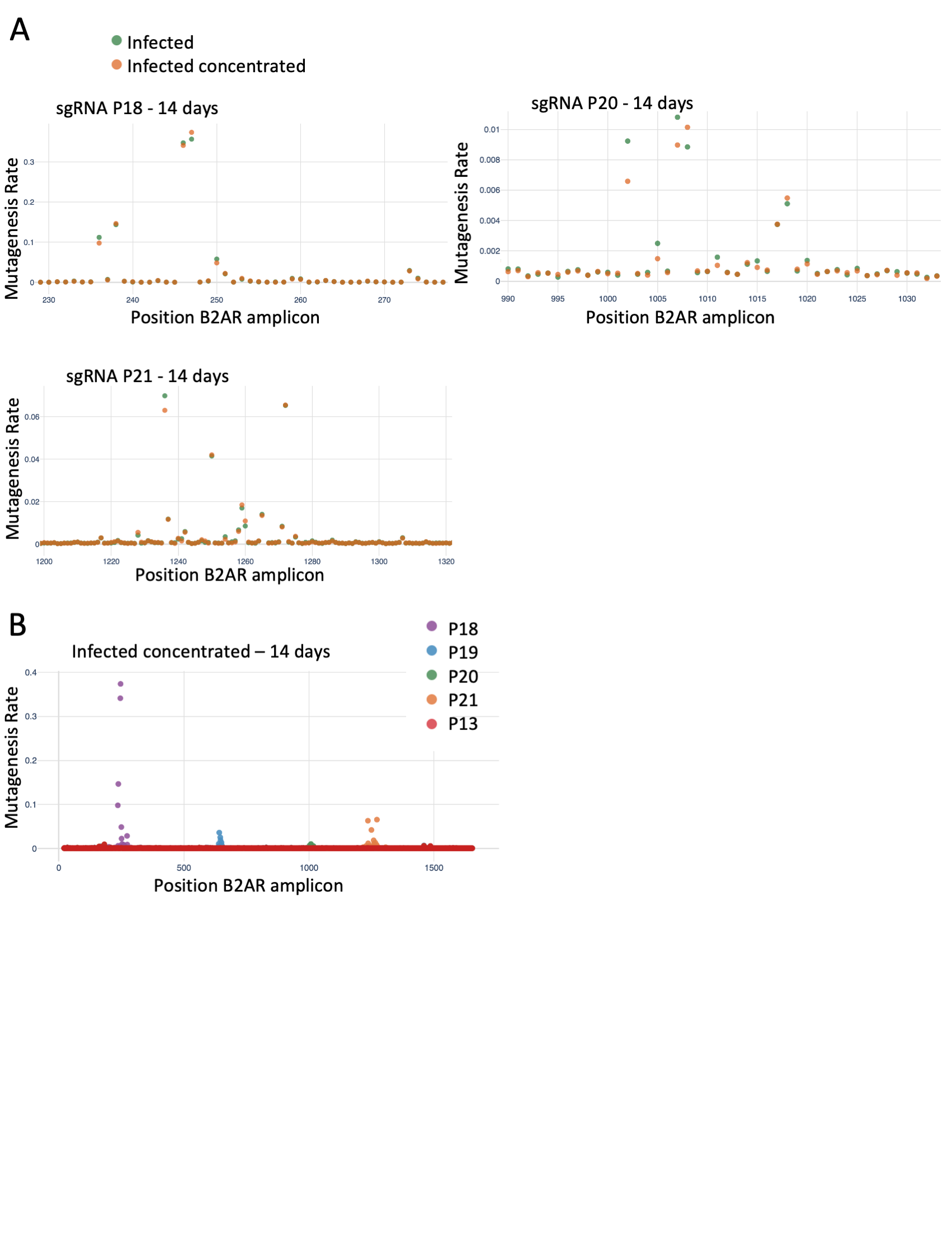

Supplement: S3 Fig — A. Mutagenesis rate per position for B2AR amplicon showing different delivery methods of the sgRNA. B. Mutagenesis rate per position for B2AR amplicon showing different sgRNA efficiencies. (TIF) [file pone.0257537.s003.tif]

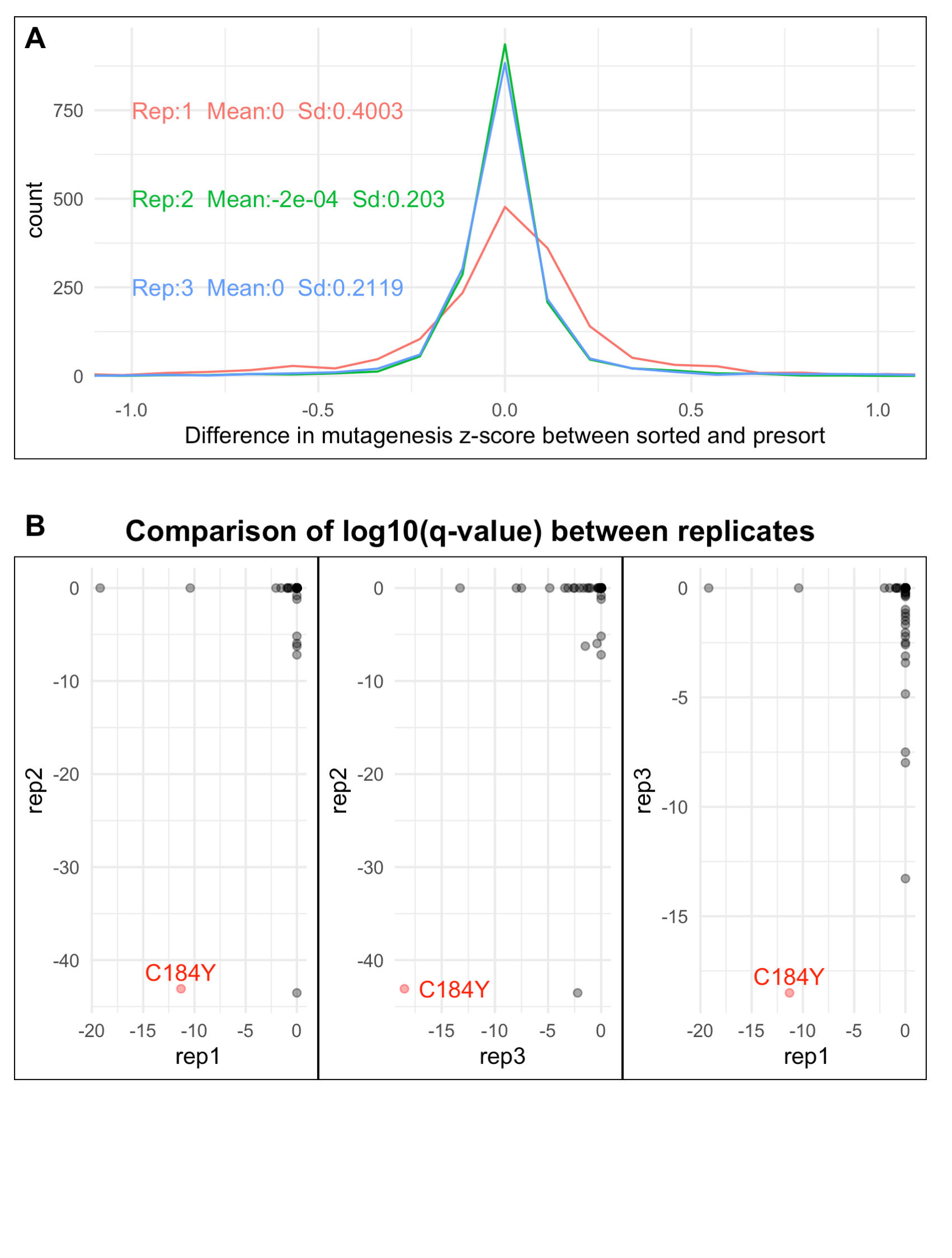

Supplement: S4 Fig — A. Distribution of the difference between the normalized mutation rates of sorted and presort samples. The mean and standard deviations of each replicate are written within the plot. B. Scatterplots showing the q-values corresponding to each B2AR position, compared across pairs of replicates (see axis labels). Note that the values displayed are log10(q-value). The single position reaching significance in all replicates is colored and labelled in red (C184Y mutation). (TIF) [file pone.0257537.s004.tif]

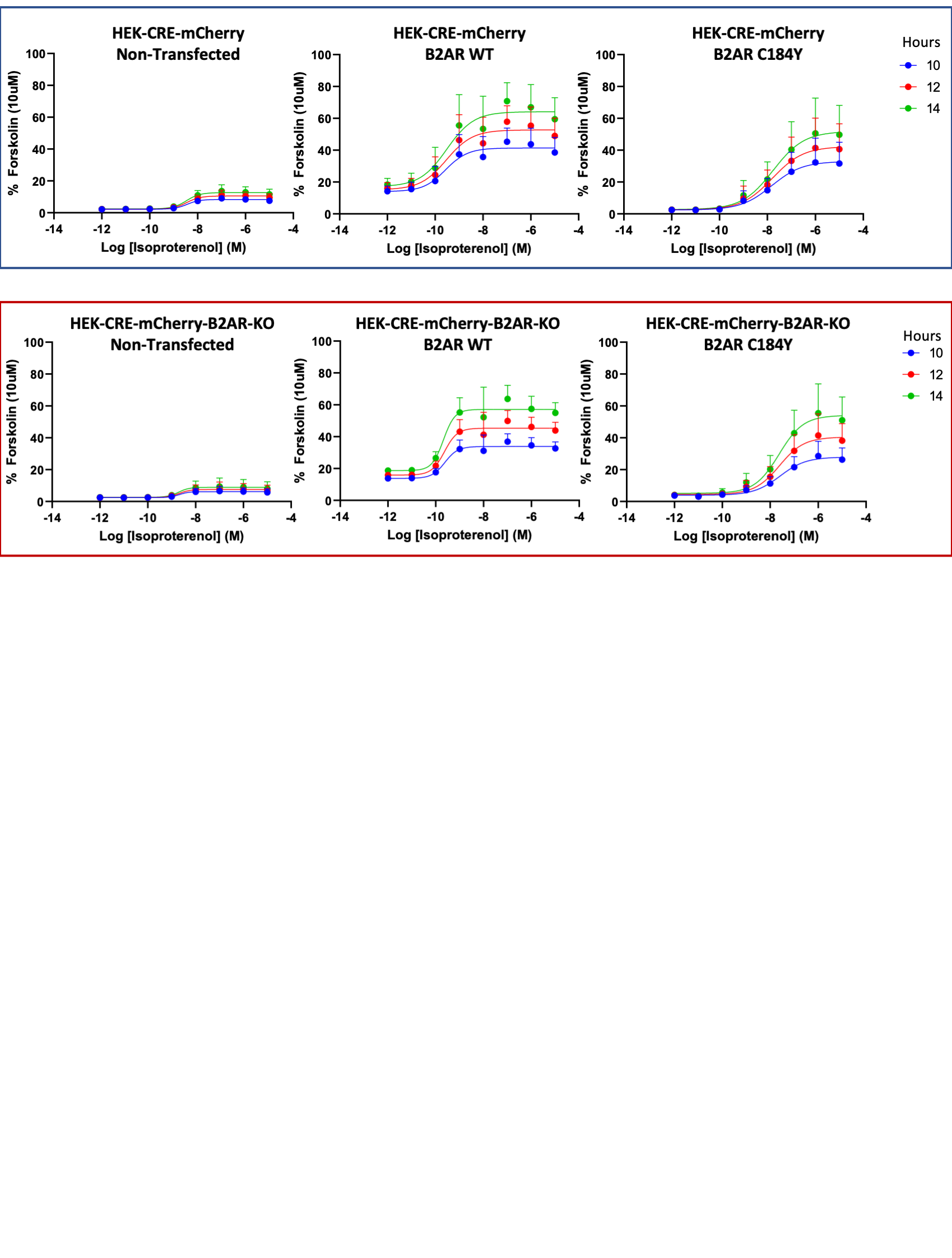

Supplement: S5 Fig — Over three time points, 10, 12, and 14 hours, post isoproterenol stimulation. A predicted, time-dependent accumulation of mCherry intensity in both reporter cell backgrounds was observed for all conditions. Isoproterenol dependent CRE-mCherry activity is expressed as a percentage of 10μM forskolin, results are plotted of mean data of N = 3 ± SEM. Data was normalized to the 14h timepoint. (TIF) [file pone.0257537.s005.tif]

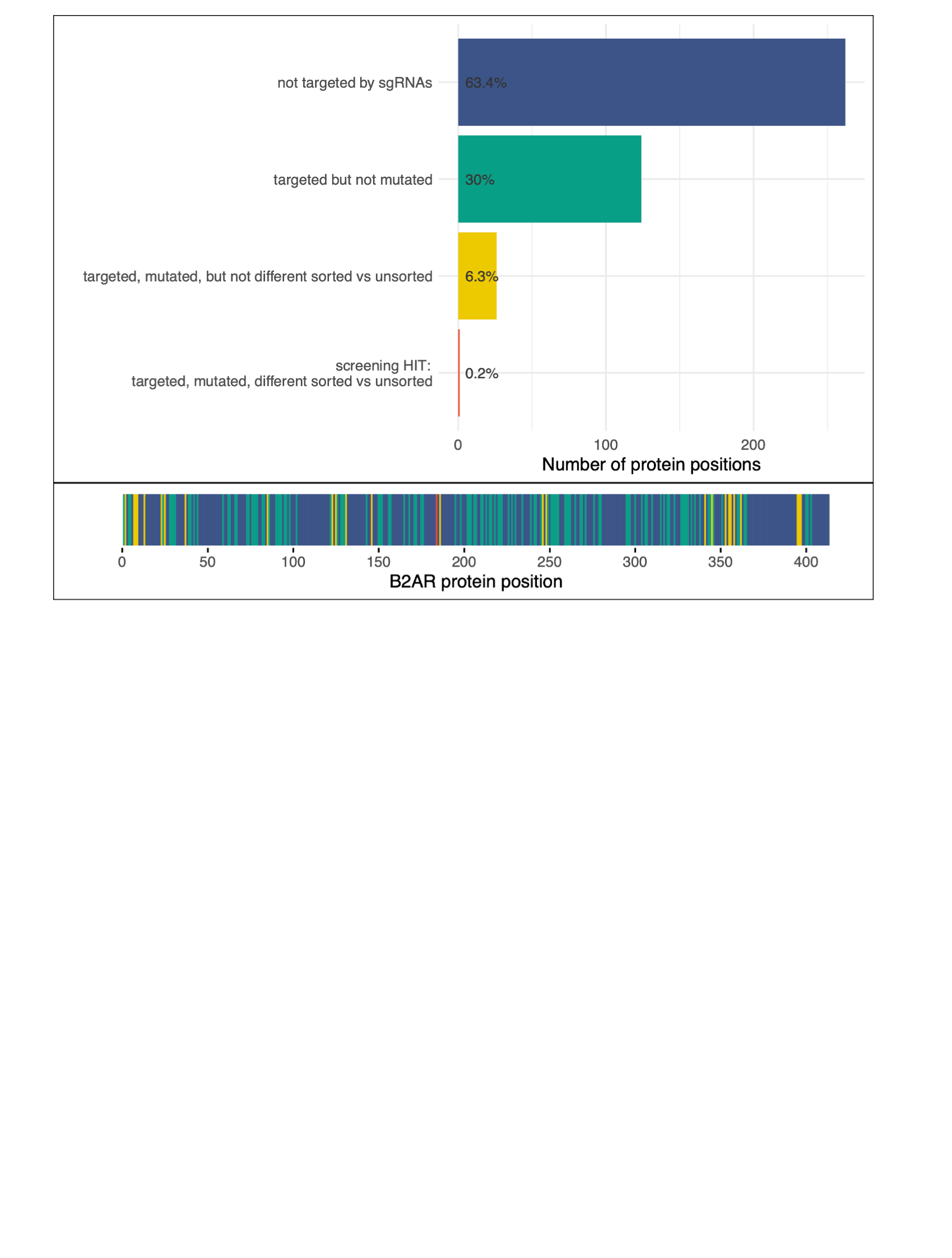

Supplement: S6 Fig — Some positions could not be targeted in the first place (blue), meaning that there were no sgRNA in the library predicted to introduce missense mutations at these codons. Others were targeted but never observed mutated (green; z-score<2 in all samples with the sgRNA library). The rest of positions were effectively interrogated by our screening, and resulted either negative (yellow) or positive hits (red; i.e., mutations reducing the B2AR response to isoproterenol). (TIF) [file pone.0257537.s006.tif]
