# Supplementary material for: Roadmap for the use of base editors to decipher drug mechanism of action
Source: PLoS One. 2021 Sep 21;16(9):e0257537. doi: 10.1371/journal.pone.0257537 (PMC8454938; doi:10.1371/journal.pone.0257537)
Supplement: S1 Table — (DOCX) [file pone.0257537.s007.docx]

S1 Table. sgRNA sequences

| **sgRNA name** | **sequence** |
| --- | --- |
| sgGFP10 | CGTCGCCGTCCAGCTCGACC |
| sgGFP1 | GGCGAGGGCGATGCCACCTA |
| sgNegCtrl1 / sgRNA P13 | GCTCAAGAACGCCTTCCCCAGTC |
| sgNegCtrl2 | TCCCCCTCAGCCGTATT |
| sgRNA P18 | AAGAAGGCGCTGCCGTTCCC |
| sgRNA P19 | TGCTGACCAAGAATAAGGCC |
| sgRNA P20 | AGCAGAACTTGGAAGATCTG |
| sgRNA P21 | GGGAATGGCTACTCCAGCAA |
