# Supplementary material for: Roadmap for the use of base editors to decipher drug mechanism of action
Source: PLoS One. 2021 Sep 21;16(9):e0257537. doi: 10.1371/journal.pone.0257537 (PMC8454938; doi:10.1371/journal.pone.0257537)
Supplement: S3 Table — (DOCX) [file pone.0257537.s009.docx]

S3 Table. Oligos to PCR genomic DNA

| **Amplicon** | **Oligo Forward** | **Oligo Reverse** |
| --- | --- | --- |
| GFP | GAGAATCGGACGGGGGTAGT | GGTCACGAACTCCAGCAGGA |
| B2AR | AAGCGGCTTCTTCAGAGCAC | CCATGCAAAGAGGAACTGAACT |
