# Supplementary material for: Roadmap for the use of base editors to decipher drug mechanism of action
Source: PLoS One. 2021 Sep 21;16(9):e0257537. doi: 10.1371/journal.pone.0257537 (PMC8454938; doi:10.1371/journal.pone.0257537)
Supplement: S4 Table — Parameters are derived from S5 Fig. (DOCX) [file pone.0257537.s010.docx]

S4 Table. Effect of B2AR-C184Y mutation on isoproterenol dependent CRE-mCherry activity over time. Parameters are derived from Fig S5.

|  | | | **10 hour** | | **12 hour** | | **14 hour** | |
| --- | --- | --- | --- | --- | --- | --- | --- | --- |
| **Cell Background** | **Plasmid** | **N** | **p[A]_50_** | **E_max_** | **p[A]_50_** | **E_max_** | **p[A]_50_** | **E_max_** |
| HEK-CRE-mCherry | Non-transfected | 3 | 8.45±0.4 | 8.36±2.4 | 8.47±0.4 | 10.54±4.3 | 8.50±0.4 | 12.69±6.1 |
|  | B2AR-wt | 3 | 8.83±0.8 | 42.48±16.0 | 8.84±0.4 | 54.05±18.7 | 8.83±0.9 | 65.8±22.4 |
|  | B2AR-C184Y | 3 | 7.75±0.2 | 32.65±25.2 | 7.68±0.2 | 42.00±30.5 | 7.64±0.3 | 51.48±35.5 |
| HEK-CRE-mCherry B2AR-KO | Non-transfected | 3 | 8.57±0.04 | 6.2±4.3 | 8.60±0.02 | 7.56±6.3 | 8.65±0.01 | 8.91±8.2 |
|  | B2AR-wt | 3 | 8.69±1.3 | 35.25±8.4 | 8.97±0.9 | 47.47±10.9 | 8.97±1.0 | 59.93±14.1 |
|  | B2AR-C184Y | 3 | 7.43±0.5 | 28.42±13.4 | 7.46±0.5 | 41.29±19.5 | 7.49±0.5 | 55.27±26.5 |
