# Supplementary material for: Roadmap for the use of base editors to decipher drug mechanism of action
Source: PLoS One. 2021 Sep 21;16(9):e0257537. doi: 10.1371/journal.pone.0257537 (PMC8454938; doi:10.1371/journal.pone.0257537)
Supplement: S1 Raw images — (PDF) [file pone.0257537.s014.pdf]

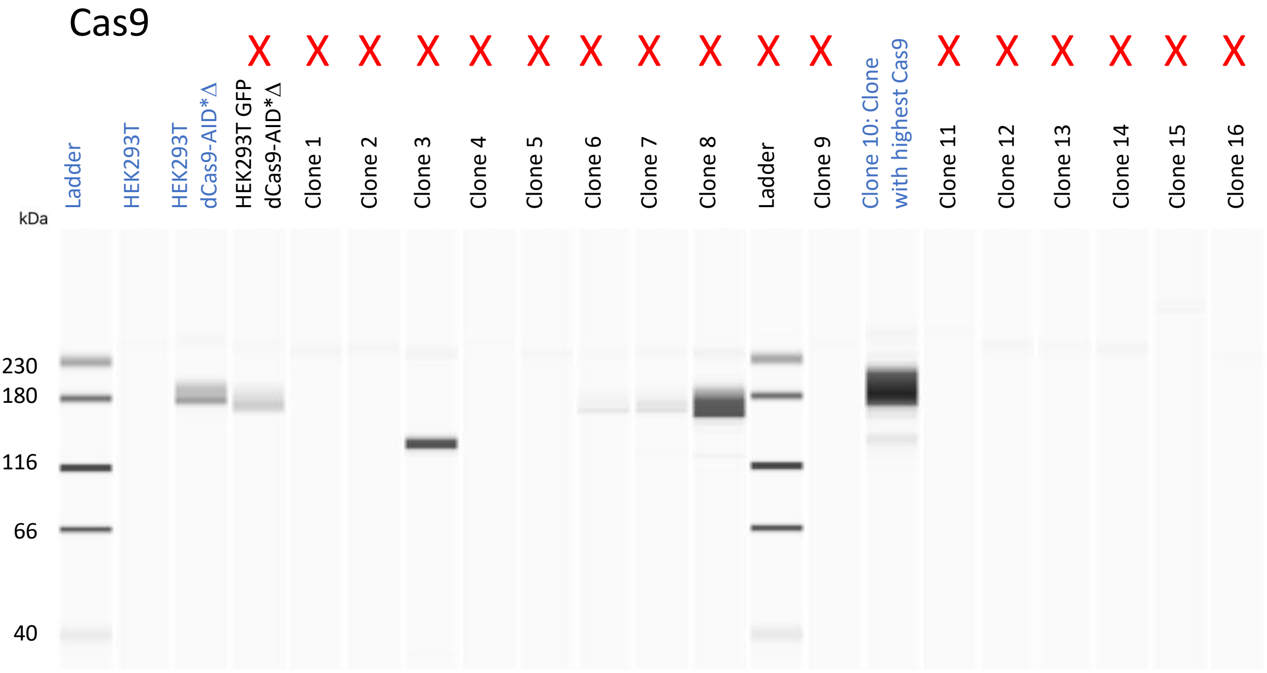

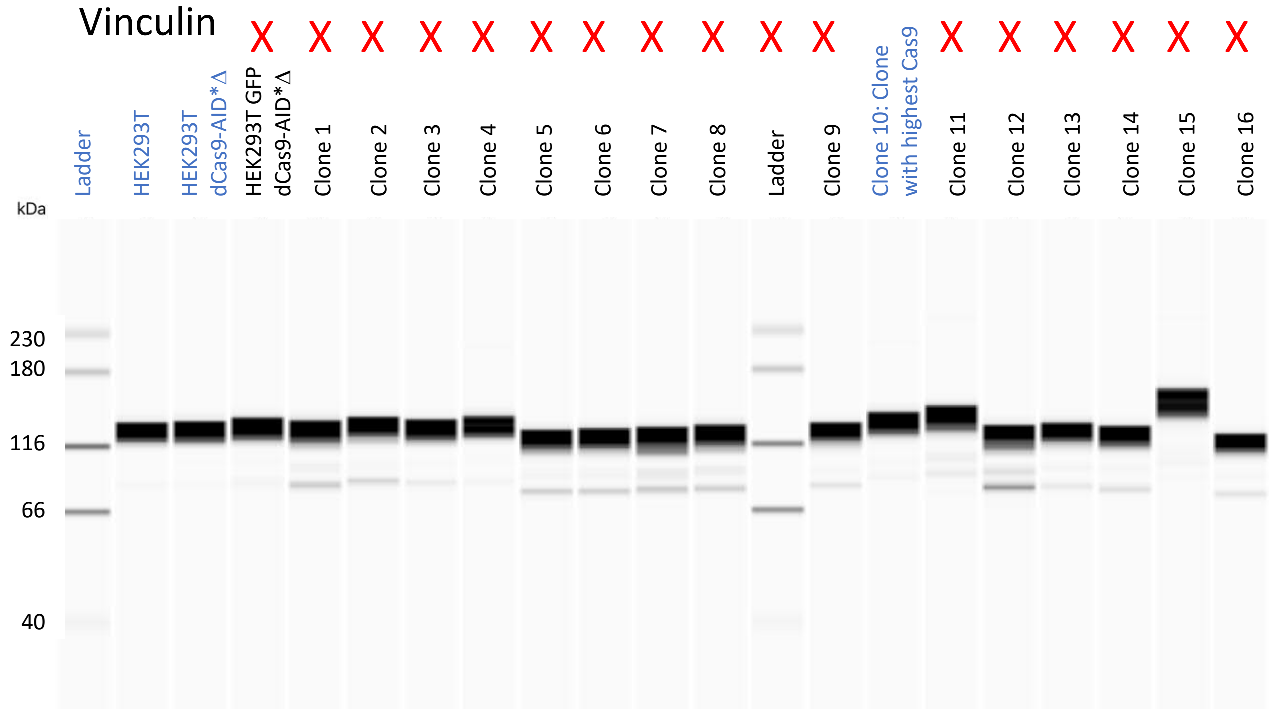

Experimental samples are indicated in blue.
Images were captured with Peggy Sue (Protein Simple).
Fig 3A and S1 Fig were generated with this blots.
